# Assessment of Genitourinary Trauma in Southeastern Iran

**DOI:** 10.5812/traumamon.11694

**Published:** 2013-10-13

**Authors:** Amene Sabzi Sarvestani, Mehdi Zamiri

**Affiliations:** 1 Department of Surgery, Imam Ali Educational Hospital, Zahedan University of Medical Sciences, Zahedan, IR Iran

**Keywords:** Urogenital System, Trauma, Epidemiology, Iran

## Abstract

**Background:**

To survey genitourinary (GU) organ injury following general trauma, we performed an epidemiologic study of urogenital injuries in trauma patients referred to our hospital (a teaching hospital affiliated with the Zahedan University of Medical Sciences).

**Objectives:**

We aimed to assess the epidemiology of urogenital system injuries in southeastern Iran.****

**Patients and Methods:**

From April 2009 to November 2011, all patients with GU injuries referred to our hospital were studied. The data including age, sex, type of injury, mechanism of trauma, and prognosis of patients was collected and analyzed.

**Results:**

From a total of 3450 patients, 66 (1.91%) had injuries of the urogenital system; 49(74.24%) were male and 17(25.75%) female. The patients’ mean age was 23 ± 12 years (range 2 to 75 years). Of these 66 patients, 61 (94.24%) had blunt trauma, and 5 (7.57%) had penetrating trauma. Motor vehicle accidents were the most common cause of trauma (63.63%). The most common injured organs were kidneys in 41 (62.12%) and the bladder in 9 (13.6%); 47 patients (71.21%) had associated intra-abdominal injuries, and 42 (63.63%) had other accompanying injuries; 23(34.84%) patients required surgical intervention. Three patients (4.54%) died due to the severity of injuries (Injury Severity Score > 12).

**Conclusions:**

In our assessment, blunt trauma including road traffic accidents were the main cause of urogenital injuries. Most patients with urogenital trauma had multiple injuries, and required a multidisciplinary approach for management.

## 1. Background

Disabilities caused by trauma has become one of the most serious public health problems in developed countries as well as countries with low total annual income (1).There is not enough data regarding the epidemiology of genitourinary trauma in different populations. In the West, one of the very few studies in this field was performed by Bariol and colleagues who reported a 1.5% incidence of GU trauma in Scotland (2). McAninch et al. showed that urogenital traumas are responsible for up to 10% of trauma admissions in the United States (3) while other studies in Iran (4) and the United Arab Emirates (5) showed a prevalence of 0.98% and 0.9%, respectively. There are few reports on genitourinary trauma in the Middle East (6-9). Furthermore, most of the available data pertain to genitourinary injuries among soldiers not civilians (7, 8). 

## 2. Objectives

The etiology of trauma differs in different populations; we sought to see how the epidemiology of genitourinary trauma varies. Thus, the aim of this study was to report the prevalence and nature of genitourinary injuries to compare with other studies.

## 3. Patients and Methods

This study was performed from April 2009 to November 2011 (a 20 month period) at our referral center for trauma patients in Sistan and Baloochestan province (a major province in southeastern Iran). Data collection was done in the emergency department. A total of 3450 trauma patients were seen. Those with urogenital injury were documented. The collected data included demographic characteristics, trauma mechanism, type of injury, severity of injury and associated injuries. The ISS was used to provide an overall score for patients with multiple injuries. Each injury was assigned an Abbreviated Injury Scale (AIS) score allocated to one of the 6 body regions (head, face, chest, abdomen, and extremities and external regions) (6). Only the highest AIS score was used in each body region. The first 3 most severely damaged body regions had their scores squared and added together to yield the ISS score.

## 4. Results

**Figure 1.**
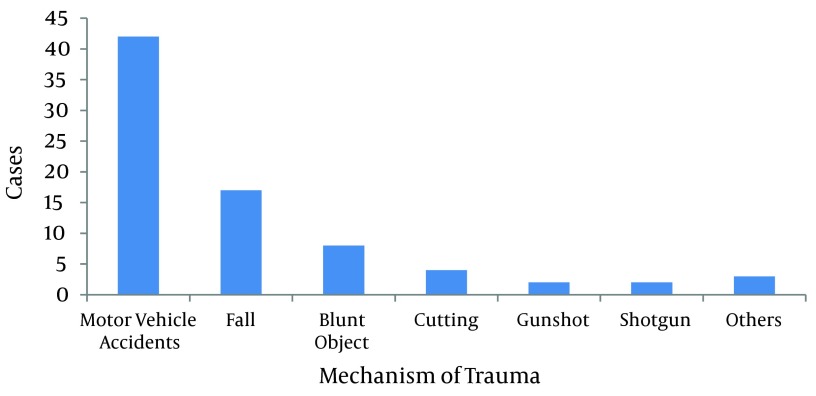
Mechanism of Trauma in Patients With Urogenital Trauma

Of 3450 trauma patients, 66 (1.91%) had injuries of the urogenital system; forty-nine (74.24%) patients were men and 17 (25.75%) women. The patients’ mean age was 23 ± 12 years (range 2 to 75 years). The associated injuries were head and neck injuries in 20 (30.3%), thoracic injuries in 12 (18.18%), abdominal-pelvic injuries in 47 (71.21%), and injury of extremities in 36 (54.54%). Of 66 patients, 61 (94.24%) had blunt trauma and 5 (7.57%) had penetrating trauma. Trauma mechanisms are showed in Figure 1. Motor vehicle accidents (MVA) were the most common cause (42 patients, 63.63%). In MVAs 18 (27.27%) were pedestrians, 12 (18.18%) passengers or driver, 9 (13.6%) motorcycle riders, 2 (3.03%) bicycle riders, and 1 (1.51%)had other mechanism of injuries. 

**Figure 2.**
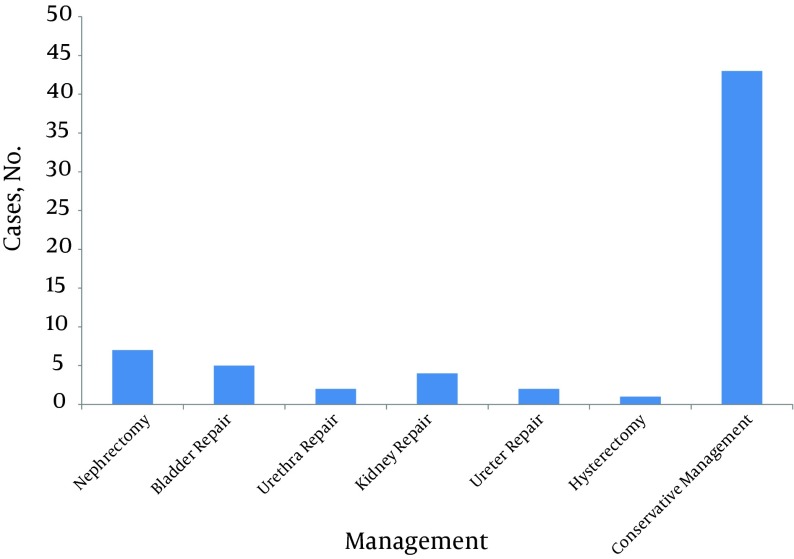
Management of Urogenital Trauma

Twenty-three (34.84%) patients required surgical management of the urogenital system (Figure 2); 19 patients (28.78%) had isolated urinary tract trauma (all survived); 3 patients (4.54%) died due to the severity of associated injuries (2 patients with kidney injury, and 1 with bladder injury).The mean ISS was 9 ± 3.1. Mild injury (ISS < 8) was seen in 22 (33.3%), moderate injury (9 < ISS < 16) in 26 (39.39%), and severe injury (ISS > 16) in 18 (27.27%) patients. 

**Figure 3.**
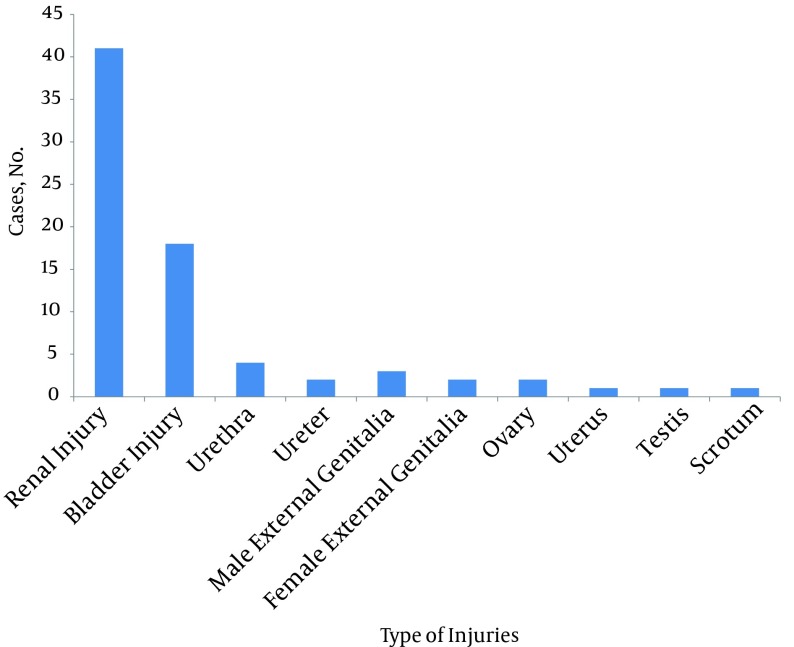
The Distribution of Urogenital Injuries

Figure 3 shows the involved genitourinary organs. Renal injuries accounted for 62.12% of all injuries. Of 41 renal injuries, 25 had renal contusion with hematuria, 6 subcapsular hematomas without laceration, and 10 renal parenchyma laceration (grades IV and V, for which 7 patients required nephrectomy). Six patients required kidney repair and the rest were treated conservatively; 9 (13.6%) had bladder injury. One bladder laceration was extra peritoneal and managed via bladder drainage. All bladder lacerations were associated with pelvic fractures. Five bladder lacerations were intraperitoneal and were repaired. Three bladder injuries were contusions and hematoma. One of four urethral injuries involved complete disruption of the membranous urethra and was associated with pelvic fracture. The other three urethral injuries were in the anterior part (two superficial contusion injuries without extravasation, and one superficial rupture with extravasation). Two ureteral injuries were managed by operative repair and stenting. One deep male external genitalia laceration was debrided and repaired. Two other male external lacerations were managed conservatively by elevation with towels and cold packs. One testicular injury was in the form of hematoma without need for exploration. One scrotal wall injury was managed via debridement and primary repair of the skin, and the other injury was in the form of second degree scrotal skin burn. A single posterior vaginal wall injury was managed with debridement and primary repair. A laceration of the labia major was managed by debridement and primary repair. Two lacerations of ovaries were treated conservatively; while uterus laceration was associated with pelvic fracture and required hysterectomy. 

## 5. Discussion

Trauma is a main cause of mortality in the ages between 1 and 44 years in the United States and some believe that it is the main cause of mortality for this age group worldwide. The rate of injuries leading to disability is 2.5 times the mortality caused by trauma (10). Wessells et al. reported 6231 renal injuries (incidence: 1.2% per 100000 population) in 523,870 patients hospitalized for trauma in 1998 and 1999 in America (11). One study in the United States of America, reported a prevalence of 2.6% for renal injuries among 14763 children (12). However, the epidemiology of overall genitourinary organ injury in trauma patients was reported less than expected. Bariol et al. studied 24666 patients with severe trauma presenting to all major Scottish hospitals from 1999 to 2002, and 362 patients were found to have genitourinary injuries (comprising 1.5% of the trauma patients) (2). Adequate data on genitourinary trauma in Iran is not available. Different sociodemographic factors affect the incidence and nature of genitourinary injuries in this important region. In a report from Iran, Salimi and colleagues reported a 0.5% incidence of genitourinary injuries from a general trauma registry (13). In another study by Salimi and colleagues, the incidence of genitourinary trauma was reported to be 0.98% (4). The prevalence of genitourinary trauma in our registry (1.91%) was higher than other reports from other centers of Iran and the Middle East (0.5% and 0.9% respectively) (4). Hammad and colleagues reported a prevalence of about 0.5% in the United Arab Emirates (5). Bariol and colleagues reported a 1.5% rate of GU trauma in Scotland (2). These differences may relate to drivers compliance considering that the most common cause of genitourinary trauma is motor vehicle accidents. Although in some studies the most common cause of urogenital injury was firearm injury (14), and this is increasing in some African countries. The most common cause of urogenital injury in our study was blunt trauma from MVAs. This may be due to the low rate of firearms being available in our country. Kidney injury was the most common organ injured, and nephrectomy was the most common surgical management. As in the study conducted by Franco and colleagues (15), the bladder was the second injured organ. The characteristics of injured patients were comparable to those in the literature, and the number of men admitted to the hospitals was nearly 2.88 times higher than women (4, 13, 16), and most patients were in the 3rd decade of life (4, 13, 16, 17). In a study by Hassan et al. after the Bam earthquake, a different pattern of urogenital trauma was prevalent with urethral injury being the most common trauma (18). Hemodynamically unstable patients are more likely to have multiple injuries. Injuries to the kidney and bladder, associated with other injuries (higher ISS) result in a higher mortality rate. However, it seems that there is no association between the severity of isolated urogenital trauma and the outcome in these patients, a finding that has been previously reported (19, 20). Thus, patients with multiple traumas require a multidisciplinary approach, preferably by an experienced emergency surgeon (20, 21). Although all trauma patients with isolated injuries to the urogenital system survived in this study, management should not be delayed (21, 22). These injuries may lead to urogenital dysfunction; and neglect may cause serious sequelae (23). Urogenital injuries comprise a low percentage of injuries of trauma patients, and blunt traumas including road traffic accidents are the main cause of urogenital injury. This study shows a need to enforcing safety laws for prevention of MVAs. As most patients with urogenital trauma have multiple injuries, they require a multidisciplinary approach; disregarding these injuries may lead to serious consequences.
